# Long-Term Safety of Growth Hormone Therapy: Still a Controversial Issue

**DOI:** 10.3389/fendo.2012.00115

**Published:** 2012-09-19

**Authors:** Stefano Cianfarani

**Affiliations:** ^1^Department of Systems Medicine, Tor Vergata University and Molecular Endocrinology Unit-Bambino Gesù Children’s HospitalRome, Italy

Substantial experimental evidence supports the involvement of growth hormone (GH) and, especially, its main effector insulin-like growth factor-I (IGF-I) in the development, growth, and dissemination of several types of cancer (Cianfarani and Rossi, 1997; Holly et al., 1999, Renehan et al., 2004; Clayton et al., 2011). However, although the data from *in vitro* and *in vivo* studies are robust, the evidence in humans is weak (Clayton et al., 2011).

Growth hormone extracted from human pituitaries (hGH) became available for therapy of GH deficient children in early 1960s. In 1985, following the reports of an association of this treatment with the occurrence of Creutzeldt–Jakob disease, human GH was banned and replaced by recombinant hGH (rhGH). Since then, due to the unlimited supply of this molecule, the indications for rhGH have greatly expanded worldwide, now including childhood and adult GH deficiency, Turner syndrome, chronic renal failure, small for gestational age (SGA), Prader–Willi syndrome, Noonan syndrome, SHOX deficiency, idiopathic short stature (ISS), and AIDS wasting. Despite a so long time of use in so many different clinical conditions, rhGH has rarely been associated with the occurrence of serious side effects. However, longitudinal studies aimed at evaluating the health of adult subjects treated with rhGH during childhood are few and often not independent of Pharmaceutical Companies. Nevertheless, an association of hGH or rhGH treatment with a higher incidence of cancer (Swerdlow et al., 2002) and type 2 diabetes (Cutfield et al., 2000; Child et al., 2011) has been reported.

The recent publication of two studies stemming from SAGhE (Safety and Appropriateness of GH treatments in Europe) European consortium, which was started in 2009 and includes eight countries (France, Sweden, Belgium, the Netherlands, Switzerland, Germany, United Kingdom, and Italy), has generated confusion and controversy rather than shedding light on this delicate matter (Carel et al., 2012; Sävendahl et al., 2012). The French study reported a significant increase in both all-cause mortality and specific mortality for bone tumors or cerebral hemorrhage in a cohort of about 6500 young adult subjects treated with rhGH during childhood for GH deficiency, SGA, or ISS (Table 1). On the basis of these findings, in 2010 the EMA’s Committee for Medicinal Products for Human Use (CHMP) launched a warning aimed at narrowing the dose range used in the different indications approved in Europe and started a review of all available data to assess the risk-benefit balance. In contrast to the French data, the other study involving patients from Sweden, The Netherlands, and Belgium, reported not a single case of death from cancer or cerebrovascular disease in about 2500 subjects with the same diagnoses (Table 1).

Table 1**Preliminary data reported from SAGhE (Safety and Appropriateness of Growth hormone treatments in Europe) project**.

**Table:** 

France	Neoplasms	Observed	Expected	SMR (95% CI)
	All-cause	7	6.89	1.02 (0.41–2.09)
	Malignant neoplasm	3	0.60	5.00 (1.01–14.61)
	of bone and articular			
	cartilage			
	Cerebrovascular	4	0.76	5.29 (1.42–13.55)
	diseases

**Table:** 

Sweden, The Netherlands, Belgium	Cause of Death	Number of deaths
	Traffic accident	5
	Accident	2
	Suicide	4
	Homicide	1
	Poisoning	4
	Pneumonia	1
	Other Endocrine Dysfunction	1
	Primary cardiomyopathy	1
	Immune deficiency	1
	Coagulation defect	1
	Cancer	0
	Cerebrovascular diseases	0

*Data are expressed in Standardized Mortality Ratio (SMR) for France, and absolute number of deaths for Sweden, The Netherlands and Belgium*.

Both studies show a series of potential biases such as the relative small sample size, the low event rate, the lack of an untreated control group available for comparison, and the lack of reference data in the age groups including height. In addition, it is hard to explain the opposite results. Differences in genetics, environment, or, more simply, confounding factors might account for the discrepancy between the two SAGhE sub-studies.

Due to the large number of individuals treated with rhGH nowadays, it is urgent to obtain definitive data on the safety of this therapy. The SAGhE project is due to finish in November 2012 and the analysis of the whole cohort (about 25,000 subjects) will provide more complete and robust data. These two preliminary SAGhE reports have undoubtedly the merit to stress the importance to replicate these surveys in different contexts, and to provide the methodological background for a joint effort involving all the stakeholders (physicians, regulatory agencies, pharmaceutical companies, patients, and their families), to invest resources and expertise in setting up an international task force aimed at addressing this still controversial issue (Rosenfeld et al., 2012).
